# Perceptions about Graves’ Disease: A qualitative study on reports of patients in Euthyroidism and with Ophthalmopathy under a Brazilian university specialized outpatient follow-up

**DOI:** 10.1192/j.eurpsy.2023.1616

**Published:** 2023-07-19

**Authors:** E. R. Turato, D. E. Zantu-Wittmann, J. C. A. Reis, L. S. Valladão

**Affiliations:** Laboratory of Clinical-Qualitative Research, State University of Campinas, Campinas, Brazil

## Abstract

**Introduction:**

Knowing mental representations about the phenomenon of illness and medical care allows the clinical team to have better emotional handling of their patients, with gains in greater adherence to treatments. Graves’ Ophthalmopathy is an inflammatory disease with primary involvement of the extraocular muscles and orbit, being the most frequent extrathyroidal manifestation of Graves’ Disease. Many patients have psychological status changes even after successful treatment of hyperthyroidism, especially when the disfiguring signs of ophthalmopathy are predominant. An understanding of the symbolic aspects linked to this condition help doctors and nurses to have a relationship more harmonious with them.

**Objectives:**

To interpret emotional meanings in reports of patients with euthyroidism and with ophthalmopathy under follow-up at a specialized university endocrinology outpatient follow-up, discussing contradictions perceived between a stigmatized body and clinical-laboratory euthyroidism.

**Methods:**

Clinical-Qualitative design of Turato. Data was collected using Semi-directed interviews with open-ended questions in-depth, carried out with patients at a university hospital specialized outpatient service in South-eastern Brazil. The interview material was audio-recorded and fully transcribed. The interviews were treated by Clinical-Qualitative Content Analysis described by Seven Steps’ Faria-Schützer. It is based on psychodynamic concepts from the Medical Psychology theoretical framework, whose main author is Michael Balint. The sample was closed by the Theoretical Saturation of Information studied by Fontanella and cols. The finding validation has occurred by peers at the Laboratory of Clinical-Qualitative Research, State University of Campinas, San Paolo.

**Results:**

The sample was composed by 10 patients. From the search of nuclei of meanings in the reports, four categories of analysis were constructed: 1) “No, this is not normal, I must have cancer”: psychodynamics of the doctor-patient relationship in Graves’ Disease; 2) Types of illness according to their manifestations and auto-perception: silent illness and non-silent ones; 3) “The eyes are everything”: the impacts of the disfiguring alterations of ophthalmopathy; 4) The contradiction perception between clinical and laboratory normality the stigmas of ophthalmopathy.

**Conclusions:**

The patients with severe exophthalmos, maintained emotional distress despite being euthyroid, manifested by various emotional meanings reported in the interviews. The clinical-laboratory diagnosis of Graves’ Disease alone is not sufficiently capable of responding to the psychological demands of the patients. Proper listening to emotional symbolic meanings attributed by patients can help physicians and nurses in handling this setting

**Disclosure of Interest:**

None Declared

